# In vitro antibacterial activity of *Morinda citrifolia* extracts against eight pathogenic bacteria species

**DOI:** 10.1371/journal.pone.0313003

**Published:** 2024-10-30

**Authors:** Frederick Obeng-Boateng, Stephen Wilson Kpordze, Francis Addy

**Affiliations:** 1 Faculty of Biosciences, Department of Biotechnology and Molecular Biology, University for Development Studies, Tamale, Ghana; 2 Department of Molecular Biology and Biotechnology, Pan African University Institute for Basic Sciences, Technology and Innovation (PAUSTI), JKUAT-Juja Campus, Juja, Kenya; 3 One Health Laboratory, Spanish Lab Complex, University for Development Studies, Tamale, Ghana; Universidad Autonoma de Chihuahua, MEXICO

## Abstract

Medical professionals continue to face a severe issue with the evolution of resistance to conventional antibiotics. The search for new novel compounds from plants has been proven to be the alternative solution. *Morinda citrifolia* is used traditionally for the treatment of infectious diseases. The present study investigates the antibacterial properties of *M*. *citrifolia* root, leaf, and fruit (fresh, dried, and fermented) extracts on three-gram-positive and five-gram-negative bacteria. The plant parts were processed and extracted in distilled water and ethanol (60%, 80%, and absolute (100%)). The antibacterial activities of the extracts were assessed in vitro using the agar well diffusion method, with Ciprofloxacin serving as the positive control. All the tests were conducted three times to obtain the average value of inhibition zones. Overall, root extracts showed the most significant antibacterial activity, followed by dried fruit, fermented fruit extract, fresh fruit, and the least leaf extract. Using one-way ANOVA and Tukey’s post-hoc tests, the statistical analysis revealed significant differences in antibacterial activity among the extracts and solvent concentrations. The 100% ethanol extracts had significantly higher zones of inhibition compared to the other solvents. The most inhibitory activity was against *Campylobacter* spp. (21.33±1.80) for the 80% ethanol root extract. All the extracts of *M*. *citrifolia* were found to exhibit moderate antibacterial activity against all the bacteria pathogens. However, *Enterococcus faecium*, *Campylobacter* spp., and *Bacillus cereus* were most sensitive to all the plant extracts while *Shigella* spp. and *Klebsiella* spp. showed resistance to most extracts. This observed difference is significant for each strain extract depending on the bacteria strain and the type of solvent extract (p < 0.001). The findings indicate a promising antimicrobial potential of *M*. *citrifolia* extracts.

## 1.0 Introduction

The scientific community continues to face severe challenges due to the current issue of growing number of multi-drug resistance (MDR) microorganisms [1]. The problem is made worse by the decline efficacies of synthetic medications and associated toxicity [2]. Antibacterial resistance is becoming a bigger issue and the outlook for the use of antimicrobial drugs in the future is still uncertain. The increasing prevalence of antibiotic-resistant bacteria is a global health concern. According to the World Health Organization (WHO), more than 700,000 people die annually from drug-resistant infections. Guided by the resulting impact on human health, antimicrobial resistance was included as one of the top ten threats to global health in 2019 by WHO [3]. By the year 2050, it is predicted that without new and better treatments, antimicrobial-resistant-related deaths will cost the world one hundred trillion dollars and result in 10 million deaths annually [4]. Resistance has been developed in varied strains of microorganisms due to the misuse and overuse of synthetic medications [5]. A typical example is the superbug, Methicillin-resistant *Staphylococcus aureus* (MRSA), which exhibits resistance to methicillin and other beta-lactam antibiotics, including penicillin and cephalosporins [6].

Continued research into developing novel drugs, whether synthetic or natural, is necessary these days and will be instrumental in the future [7]. Due to this, increased emphasis has now been placed on the use of plant materials as a source of medicines for a wide range of human ailments [8,9]. In addition, attention has been diverted toward the search for novel compounds from plants over the years. These phytoactive constituents, either unaided or in combination with antibiotics may be an effective approach to dealing with global antimicrobial resistance [10]. Since herbal remedies have been successful in curing ailments for decades, this suggests that viruses, bacteria, and fungi may be less able to adjust to a regime of plant-based antimicrobial [11].

The plant *Morinda citrifolia*, usually known as Noni in Southeast Asia, is a crucial medicinal plant with a wide variety of phytochemical components, such as terpenoids, flavonoids, and alkaloids, that are noted for antibacterial effects [12,13]. In some East Asian cultures, various parts of *M*. *citrifolia* are traditionally utilized in the treatments of headaches, burns, arthritis, and even conditions associated with diabetes, hypertension, and tuberculosis [14]. It has also been suggested to have antimicrobial properties as about 200 phytochemicals were identified and isolated from various portions of the plant [15]. The compounds found in the various plant structures of *M*. *citrifolia* can exhibit antibacterial effects on a wide range of bacterial species. Secondary metabolite compounds such as flavonoids, terpenoids, alkaloids, and steroids were discovered in photochemical analyses of noni fruit’s hexane and ethanol extracts [16]. Alsheikh et al [17] mentioned pentacetyl-β-D-glucopyranose and iridoid acubin as bacteriostatic compounds. These substances discovered in the pulp of *M*. *citrifolia* fruit had antibacterial effects against *Salmonella typhi*, *Escherichia coli*, *Staphylococcus aureus*, and *Shigella dysenteriae*. Data shows that *M*. *citrifolia* casual root cultures could be a suitable method for the commercial production of biotechnology-based compounds like flavonoids, rubiadin, anthraquinones, and phenolics as a means of reducing the implications of multi-drug resistance [12].

In Africa, the exploitation of the medicinal prospects of the noni is less or poorly document. While traditional herbal folklore of its use in the treatment of several ailments exist in Ghana, very little is known of the antibacterial properties of extracts of the various plant parts in Ghana. This is important to explore how the local noni plant varieties can be utilized to control endemic bacterial infections. In the present study, we determined the antibacterial properties of *Morinda citrifolia* root, leaf, and fruit (fresh, dried, and fermented) extracts on three gram-positive bacteria (*Listeria monocytogens*, *Bacillus cereus*, *Enterococcus faecium*) and five gram-negative bacteria, (*Klebsiella* spp., *Campylobacter* spp., *Vibrio cholerae*, *Yersinia enterocolitica*, *Shigella* spp.) and also evaluate the activity index of the *M*. *citrifolia* plant extracts using ciprofloxacin for further propagation.

## 2.0 Materials and methods

### 2.1 Ethics statement

The trial was conducted on fecal samples that were preserved and available for use from a previous study that isolated bacteria from human fecal samples. So, no additional ethics approval or consent was required. The study was approved by the Department of Biotechnology and Molecular Biology, Faculty of Biosciences, University for Development Studies, Ghana.

Field access and plant materials collection (*Morinda citrifolia*) were conducted with permission from local farmers. No formal permits were required, as farmers provided free access to their farms (private land) for sourcing the plant materials.

### 2.2 Study area and sample collection

The study was conducted in the Tamale Metropolis, Northern Region, Ghana. *Morinda citrifolia* samples (plant parts; fruit, leaves, and root) were collected from three suburbs of the city namely, Lamshegu (9.4034° N, 0.8424° W), Dungu (9.483° N, 0.850° W), and Choggu (9.433° N, 0.850° W) kept in a cool box containing ice packs, and transported to the Spanish Laboratory Complex of the University for Development Studies (UDS) for further analysis. The targeted bacteria species (*Listeria monocytogens*, *Bacillus cereus*, *Enterococcus faecium*, *Klebsiella* spp., *Campylobacter* spp., *Vibrio cholerae*, *Yersinia enterocolitica*, and *Shigella* spp.) were isolated from stored human fecal samples at the Spanish Laboratory Complex and *E*. *coli* ATCC 25922 strain was used as a control. Ciprofloxacin (CIP—5 μg) was used as a positive control and fresh and fermented fruit juice without any addictive or extraction was used as a negative control. Ciprofloxacin was selected as the reference antibiotic in this study due to its availability and its widespread use in clinical settings in Ghana.

### 2.3 Plant sample and extracts preparation

The noni plant samples (fruit, leaves, and root) were washed thoroughly in sterile distilled water (dH_2_O) twice to clean dirt around it, and surface sterilized with 20% sodium hypochlorite. To obtain fresh juice, fresh fruits were carefully chosen based on their quality and suitability for juicing. The cleaned fruits were blended into a smooth-homogeneous mixture using a sterile blender (Preethi, Preethi Kitchen Appliance Pvt Ltd, India). The fermented fruit juice was obtained using a protocol of Sina et al [18] in a natural fermentation process. Some of the blended juice was transferred into a clean sealed and sterile container. The container was sealed tightly to create an anaerobic environment, crucial for the fermentation process. The juice was left undisturbed at room temperature for 60 days which allowed for the gradual development of the desired flavors and characteristics of the fermented juice. In both cases, the resulting juice was filtered on cotton and Whatman paper and was then collected and transferred into a clean, sterile container.

Following the protocol outlined by Candida et al [19] and Liya & Siddique [20] with modifications, the fruit and root samples (excluding the leaves) were cut into pieces and spread onto a clean and sterile plate, air-dried under the sun for 48 h with a clean white cloth cover to protect it from dust. The samples were then kept in an oven at 50°C for 24 h to remove the remaining water and to dry completely. The dried samples were aseptically grounded into powder using a sterile blender (Preethi, Preethi Kitchen Appliance Pvt Ltd, India). The leaves were dried in a clean, well-ventilated room at room temperature, and spread out on clean surfaces to ensure even air circulation and moisture removal.

The plant-processed samples were extracted with distilled water, and ethanol (60%, 80%, and 100%) in 1:5 (10 g of processed plant sample: 50 ml of each solvent) ratios [21]. The choice of solvents was based on their varying polarities. In addition, water and ethanol are readily available, cost-effective, and safe for biological testing. Ten grams (10 g) of each processed sample (fresh fruit juice, fermented fruit juice, powdered fruit, leaves, and root) were measured into four sterile conical flasks. Fifty milliliters (50 ml) of each solvent (100%, 80%, 60% ethanol, and distilled water) were measured and poured into the conical flasks containing the plant samples. The 1:5 ratio of plant sample to solvent was maintained to ensure consistent extraction efficiency. The solutions were placed in an electric shaker to homogenize and ensure particles dissolved completely. The homogenate was filtered using a Laboratory test sieve (Impact Laboratory test sieve IS03310-1:2000, UK), and the filtrates were kept in an oven at 50°C for 24–36 h to dry completely till the crude extract was obtained. The dried crude extracts obtained were measured into centrifuge tubes and were used to prepare the different extract concentrations of 10%, 20%, and 40% (w/v) using sterile dH_2_O. The mixtures were then vortexed using a vortex mixer to obtain uniform suspensions and used for further tests.

### 2.4 Isolation and identification of test organisms

Fecal samples (1 g) were emulsified in 9 ml of saline solution and the suspension was serially diluted. A volume of 100 μl each was pipetted from the 4^th^ and 5^th^ level suspensions and inoculated onto separately labeled agar plates, respectively, using the spread plate method. Inoculated agar plates were incubated at 32–37°C for 16–24 h (depending on the suitable growth temperature of the organism and media used) in an incubator. Isolates (*Listeria monocytogens*, *Bacillus cereus*, *Enterococcus faecium*, *Klebsiella* spp., *Campylobacter* spp., *Vibrio cholerae*, *Yersinia enterocolitica*, *Shigella* spp., and a control (*E*. *coli* ATCC 25922) with positive characteristics on agar plates were sub-cultured by streaking method on Tryptic Soy Agar (TSA) for pure culture and incubated at 35±2°C. After incubation, isolates were confirmed using the following biochemical tests; latex agglutination test kit, Indole Test, Deoxyribonuclease (DNase) Test, Oxidase Test, Catalase Test, Triple Sugar Test, and Citrate Test.

### 2.5 In-vitro antibacterial assay

The Agar Well Diffusion Method was employed for determining the antibacterial activity of *M*. *citrifolia* (fruit, leaves, and root) following the protocol of Liya & Siddique, (2018) with slight modification. Muller Hinton Agar-MHA (Oxoid, UK) was prepared according to the manufacturer’s instructions. The medium was sterilized by autoclaving at 121°C for 15 minutes and then allowed to cool before being poured into sterile Petri dishes. To ensure standardization, the turbidity of the culture suspension was adjusted to a 0.5 McFarland standard and this was done by adjusting the density of the cultures with sterile saline solution. The standardized bacterial suspensions were uniformly spread on the solidified Muller Hinton Agar contained in plates using a sterile cotton swab and labeled respectively. A 6 mm cock borer was used to create six equidistance holes in all the inoculated agar medium containing the organisms. Afterward, 100 μl of all the extracted samples in their varying concentrations (10%, 20%, and 40%) were carefully pipetted into each of the holes to test against each of the bacteria. Fresh and fermented fruit juice were used as a negative control and were also pipetted into separate holes in the same MHA plate. A ciprofloxacin (CIP—5 μg) antibiotic disc which was used as a positive control was equally placed on the surface of the agar in plates to compare the activity index against all the organisms including the control strain (*E*. *coli* ATCC 25922). After the treatment, all the plates except for *Campylobacter* were incubated at 37 ˚C for 24 h, and the diameters of the possible zone of inhibitions were measured using a graduated ruler. However, *Campylobacter* culture suspensions were inoculated on MHA supplemented with 5% sheep blood and incubated at 42 ˚C for 24 hours under microaerobic conditions using CampyGen^TM^ (2.5 L, Oxoid). All the tests were conducted three times to obtain the average value of zones of inhibition.

### 2.6 Activity index determination

The antibacterial activity of the fruit, root, and leaves of *Morinda citrifolia* was compared with the antibiotic drug (ciprofloxacin) by calculating the activity index using the formula described by Sinha et al [22] with the mean value of the zones of inhibition.


$$Activity\;index\;(mm)=\frac{zone\;inhibition\;of\;plant\;extract}{zone\;inhibition\;of\;antibiotic\;(CIP)}$$

### 2.7 Statistical analysis

Obtained data was recorded and entered into an MS Excel 2019 worksheet. Statistical analysis was performed using IBM SPSS Statistics 27. The mean zone of inhibition of the various extracts was calculated and presented as mean ± SD (Standard Deviation) using the values recorded from the inhibition of each plant sample against all the bacteria strains. All quantitative variables showed parametric distribution; therefore, One-way analysis of variance (ANOVA) was used to compare the antibacterial activity across different extracts and solvent concentrations. Tukey’s post hoc test was used for pairwise comparison between the groups when the ANOVA test was significant. This test was selected because it compares all groups while minimizing the possibility of falsely identifying significant differences, making it suitable for multiple comparisons. The significant level was set at P ≤ 0.05. Data was further described using tables and figures showing the summary of plant samples’ antibacterial inhibitions.

## 3. Results

### 3.1 Morphological identification and isolation of organism

**Fig 1:**
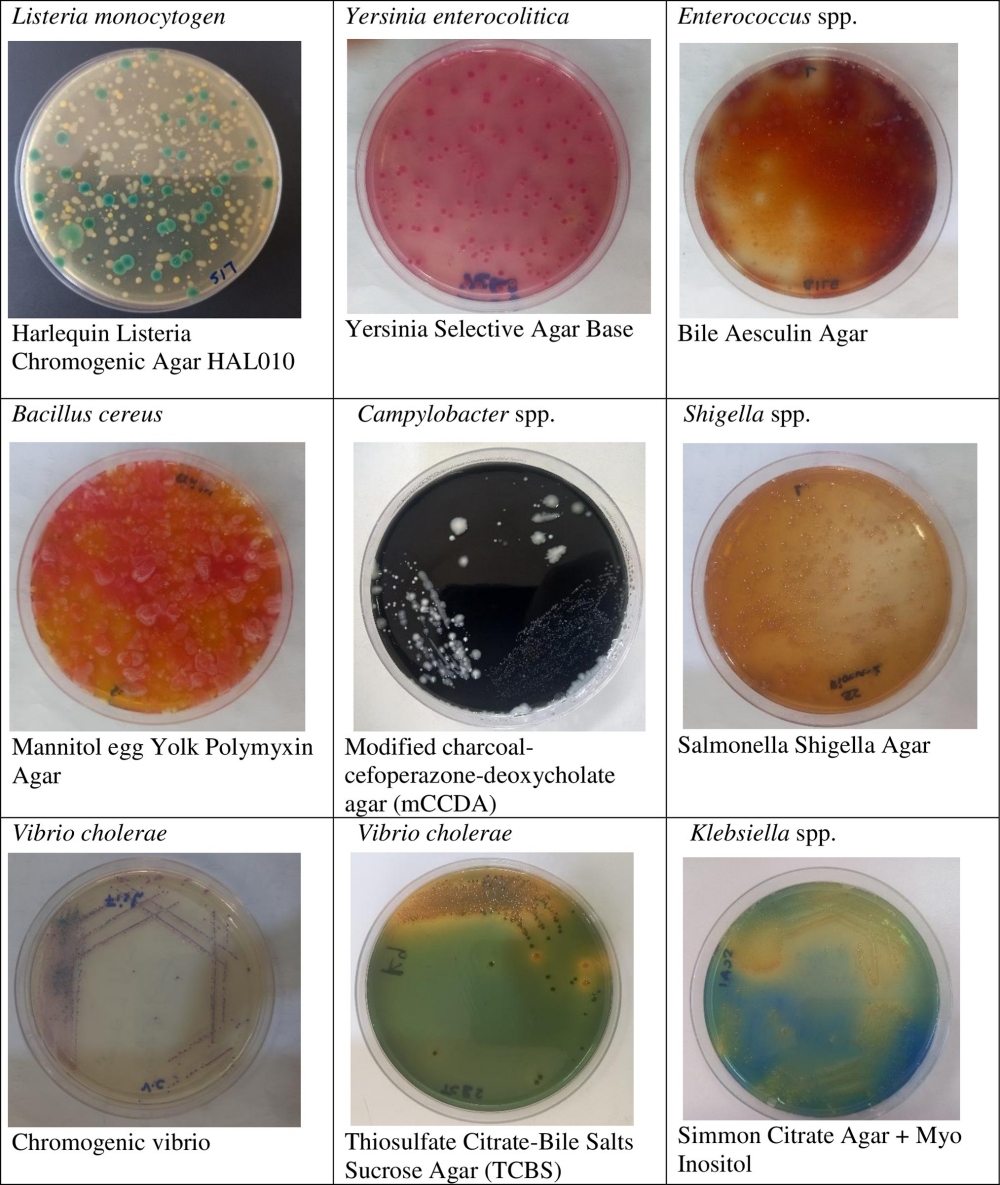
Colonies formed on prepared media after plating.

Colonies’ appearance on each agar plate is summarized in Fig 1 below guided by instructions of the media manufacturer regarding the morphological appearance of each colony on the media. This enabled distinguishing the desired colonies’ appearance from those supported by the media.

### 3.2 Antibacterial activity per zone of inhibition

**Fig 2:**
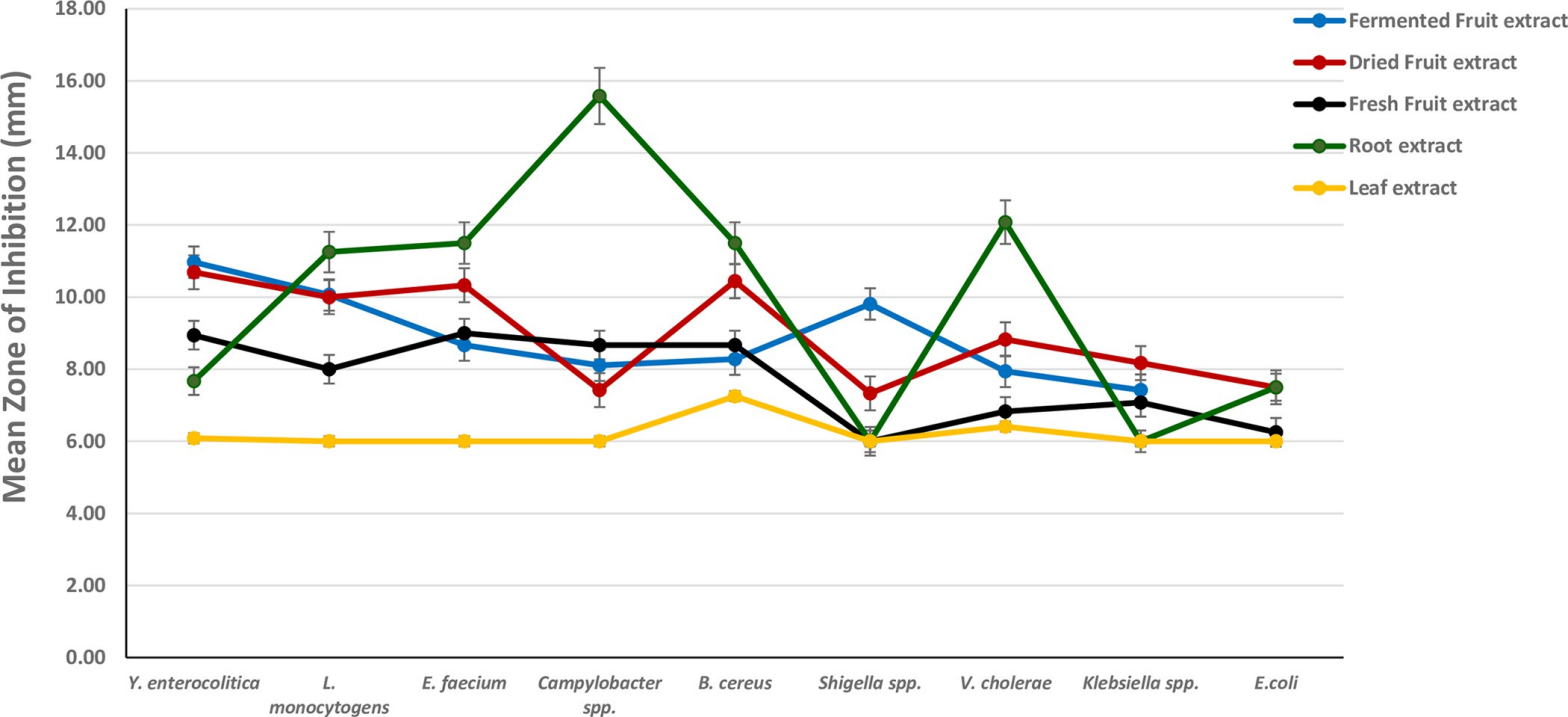
Antibacterial activity of *Morinda citrifolia* different plant part extracts across the bacterial strains.

The in vitro antibacterial activity in terms of zone of Inhibition (ZoI) ± standard deviation of the different parts of the *Morinda citirifolia* solvent extracts are represented in Table 1. below. The results obtained from the agar well diffusion assay show that the plant extracts possess antibacterial activity against the tested microorganism and there is an increasing effect on microbial growth inhibition with increasing concentration of the extract. Among the solvent extracts tested, 100% ethanol extract shows maximum inhibitory potential against the tested bacteria strains followed by 80% ethanol extract, 60% ethanol extract, and dH_2_O extract being the least. The mean ZoI for the plant extracts was found best in the root extract, followed by dried fruit, fermented fruit extract, fresh fruit, and the least with the leaf extract (Fig 2). All the average ZoI of the extracts vary significantly depending on the bacteria strain and the type of solvent extract (p < 0.001).

**Table 1:** Antibacterial activity of different extracts of *M*. *citrifolia*.

Extract/ Solvent		*Yersinia*	*Listeria*	*Enterococcus*	*Campylobacter*	*B*. *Cereus*	*Shigella*	*V*. *Cholerae*	*Klebsiella*	*E*. *coli*
**Fermented Fruit**	100% Ethanol	13.11±2.26	13.44±5.92	10.33±2.18	9±1.41	8.44±2.35	11.11±2.62	9.33±1.80	8.67±1.32	-
	80% Ethanol	12±2.06	7.33±2.00	9.67±1.00	9.11±2.42	9.67±2.83	10.33±3.04	8.44±1.88	7.67±1.80	-
	60% Ethanol	9±2.06	12.78±10.18	7.67±1.80	7.33±1.32	7.11±1.69	9.33±1.80	8.44±1.88	7.67±1.80	-
	dH_2_O	9.78±3.19	6.67±1.00	7±1.50	7±1.50	7.89±1.76	8.44±1.94	8.44±1.88	7.67±1.80	-
**Dried Fruit**	100% Ethanol	12.22±2.33	11.33±3.04	11±1.73	10.67±1.32	10.67±1.32	9±1.5	11.33±1.80	9.67±1.00	10±0.00
	80% Ethanol	11.89±1.62	10.67±2.25	11±0.87	7±1.50	11.67±3.61	7.33±0.50	11.33±2.65	9±0.00	8±0.00
	60% Ethanol	10.33±1.32	9.67±1.80	10.33±1.32	6±0.00	10.78±1.33	7±0.00	6.67±1.00	8±0.00	6±0.00
	dH_2_O	8.33±1.12	8.33±0.50	9±0.87	6±0.00	8.67±1.32	6±0.00	6±0.00	6±0.00	6±0.00
**Fresh Fruit**	100% Ethanol	10.78±2.33	9.33±2.00	10.33±2.179	9.67±1.00	9.67±2.65	6±0.00	9.33±2.00	8.±1.73	7±0.00
	80% Ethanol	9±1.73	8±1.73	9.33±1.32	10.33±2.00	9.33±1.32	6±0.00	6±0.00	7.67±1.32	6±0.00
	60% Ethanol	10±3.46	8±0.87	9±0.87	7.3±0.50	9±1.732	6±0.00	6±0.00	6.67±0.50	6±0.00
	dH_2_O	6±0.00	6.67±0.50	7.33±1.32	7.33±1.32	6.67±0.50	6±0.00	6±0.00	6±0.00	6±0.00
**Root**	100% Ethanol	9.67±1.32	10.67±0.50	11±0.00	11.67±0.50	10±0.00	6±0.00	10.67±0.50	6±0.00	6±0.00
	80% Ethanol	8±0.00	12.67±0.50	13±0.87	21.33±1.80	12.33±0.50	6±0.00	13.33±1.80	6±0.00	12±0.00
	60% Ethanol	7±0.00	12±0.87	12.67±0.50	18.67±3.61	13.33±1.00	6±0.00	14±0.87	6±0.00	6±0.00
	dH_2_O	6±0.00	9.67±1.32	9.33±0.50	10.67±1.00	10.33±0.50	6±0.00	10.33±0.50	6±0.00	6±0.00
**Leaf**	100% Ethanol	6±0.00	6±0.00	6±0.00	6±0.00	9.33±1.32	6±0.00	7.67±1.00	6±0.00	6±0.00
	80% Ethanol	6±0.00	6±0.00	6±0.00	6±0.00	7±0.00	6±0.00	6±0.00	6±0.00	6±0.00
	60% Ethanol	6±0.00	6±0.00	6±0.00	6±0.00	7±0.00	6±0.00	6±0.00	6±0.00	6±0.00
	dH_2_O	6.33±0.50	6±0.00	6±0.00	6±0.00	6±0.00	6±0.00	6±0.00	6±0.00	6±0.00
**Control (Fresh Juice)**		6±0.00	6±0.00	6±0.00	6±0.00	6±0.00	6±0.00	6±0.00	6±0.00	6±0.00
**Control (Fermented Juice)**		10.67±0.58	6±0.00	16.67±0.58	8.67±0.58	6±0.00	9.67±0.58	9.00±0.00	6±0.00	
**Ciprofloxacin (CIP)**		31.67±1.53	23.67±1.16	25.33±1.16	27.67±1.16	25.67±0.58	26±1.00	34±1.00	24.33±1.16	26±1.00

KEY: Values are Mean zone of inhibition (mm)± standard deviation, (-) means no antibacterial activity of the extract was carried on against the bacteria. Significant difference (p < 0.001)

The maximum inhibition was recorded against *campylobacter* spp. and worked best with the 80% ethanol root extract (21.33±1.80). The root solvent extracts worked effectively against almost all bacteria strains including the control (*E*. *coli*) but *Shigella* spp. and *Klebsiella* spp. showed no inhibition for all the root solvent extracts (Table 1; Fig 2). When compared with the mean ZoI of the antibiotic, the root extracts worked midway equal to the antibiotic. The fermented fruit extracts had inhibition against all the tested bacteria strains (Table 1). The highest and the lowest activities for the fermented fruit solvent extracts were both recorded against *Listeria monocytogens* with the highest generated by the 100% ethanol extract (13.44 ± 5.92) and the lowest by dH_2_O (6.67±1.00). The highest inhibition of the Dried fruit extract was produced against *Yersinia enterocolitica* 100% ethanol extract (12.22 ± 2.33). No inhibition was recorded against *Shigella*, *V*. *cholerae*, *Klebsiella*, and *E*. *coli* by the dried fruit distilled water extract. The Fresh fruit extracts worked best against *Enterococcus faecium*, *Yersinia enterocolitica*, *Bacillus cereus*, and *Campylobacter* respectively. However, *Shigella* spp. did not show any inhibition for all the solvents extract from the fresh fruit. The Leaf extract worked poorly in the present studies as most of the leaf solvent extracts did not show inhibition against most of the bacteria species. No inhibition of the leaf extract was recorded for *Listeria monocytogens*, *Enterococcus faecium*, *Campylobacter*, *Shigella*, *Klebsiella*, and the control (*E*. *coli*). The antibiotic drug Ciprofloxacin (CIP) had an inhibition zone for all the bacteria strains including the control (*E*. *coli*) (Table 1). Also, the control (Fresh fruit juice) did not show any inhibition for all the bacteria strains while the fermented fruit juice control had inhibition on almost all the extracts except for *Klebsiella* and *L*. *monocytogens* (Table 1). Almost all of the extracts were effective against all bacterial pathogens (Fig 5); however, *B*. *cereus*, *Campylobacter*, and *E*. *faecium* were found to be most sensitive to all plant extracts whereas *Klebsiella*, *Shigella*, and the control (*E*. *coli*) had the least inhibition diameter recorded against them.

**Fig 3:**
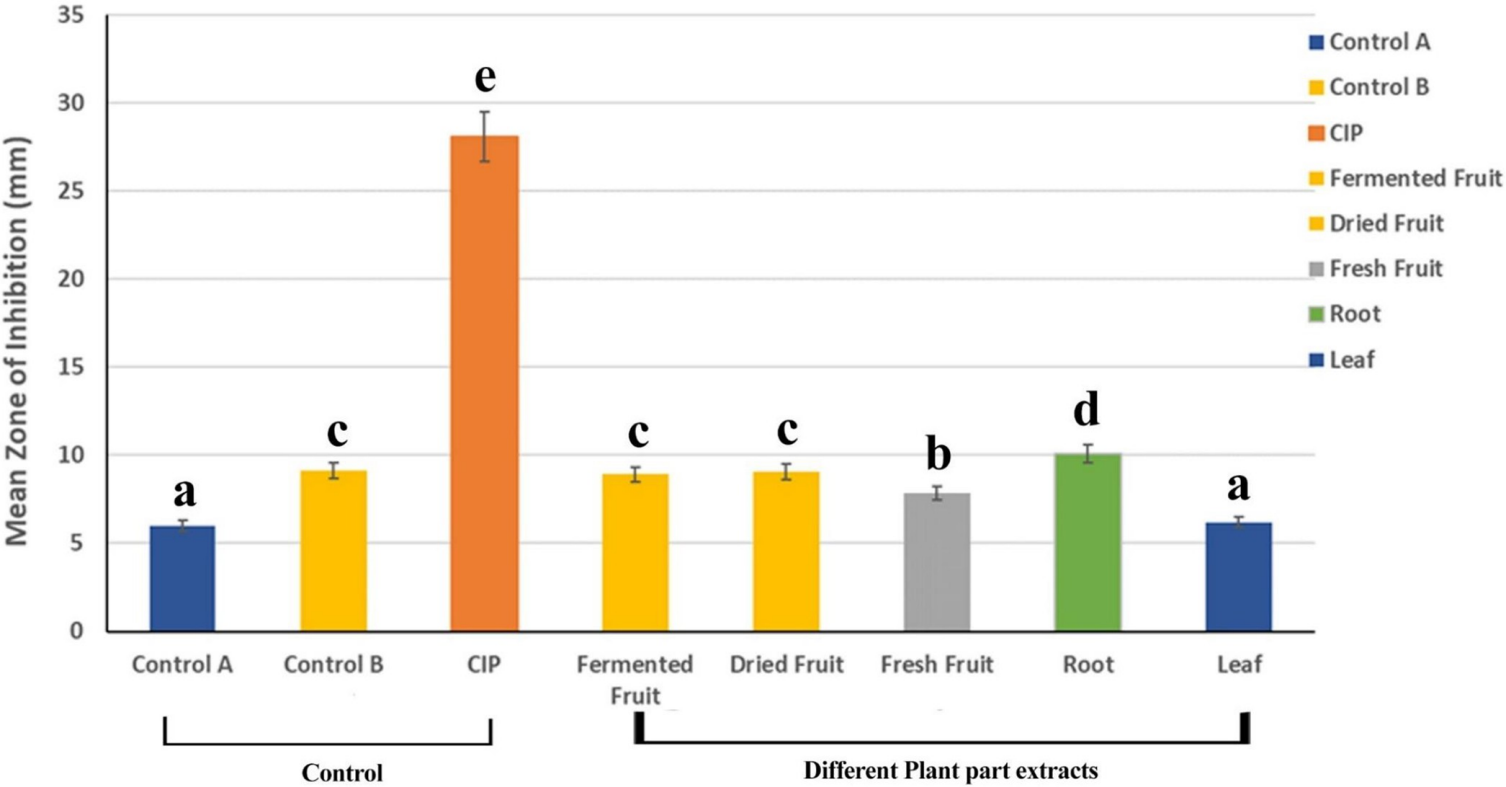
Tukey’s Post Hoc Pairwise Comparisons of Mean zone of inhibition across different extracts and 95% confidence interval of tested groups. Groups that do not share a letter are significantly different (a, b, c, d, e—Significant groups). KEY: Control A–Fresh fruit juice, Control B–Fermented fruit juice.

**Fig 4:**
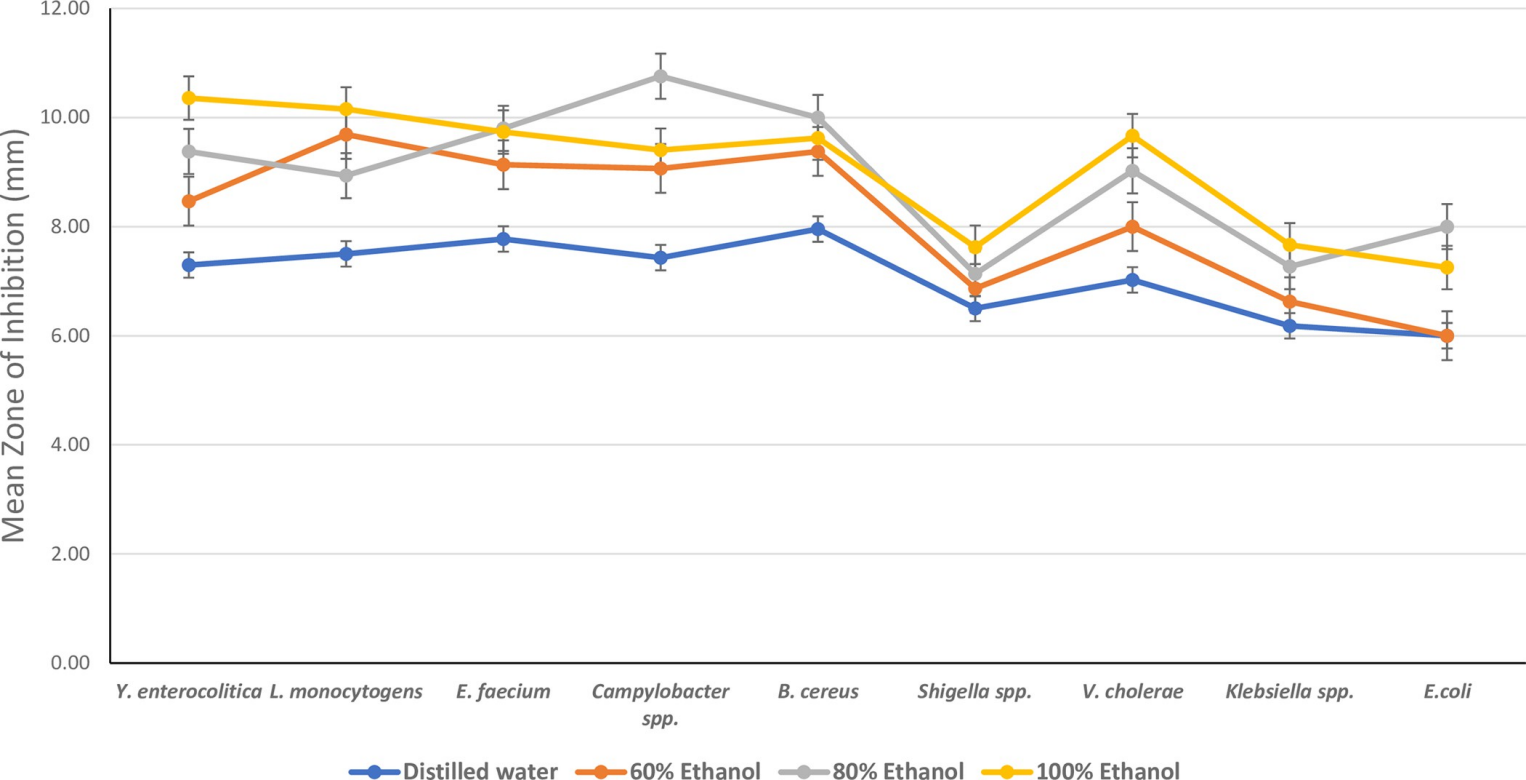
Antibacterial activity of *Morinda citrifolia* extract in different solvent concentrations against the bacterial strains.

**Fig 5:**
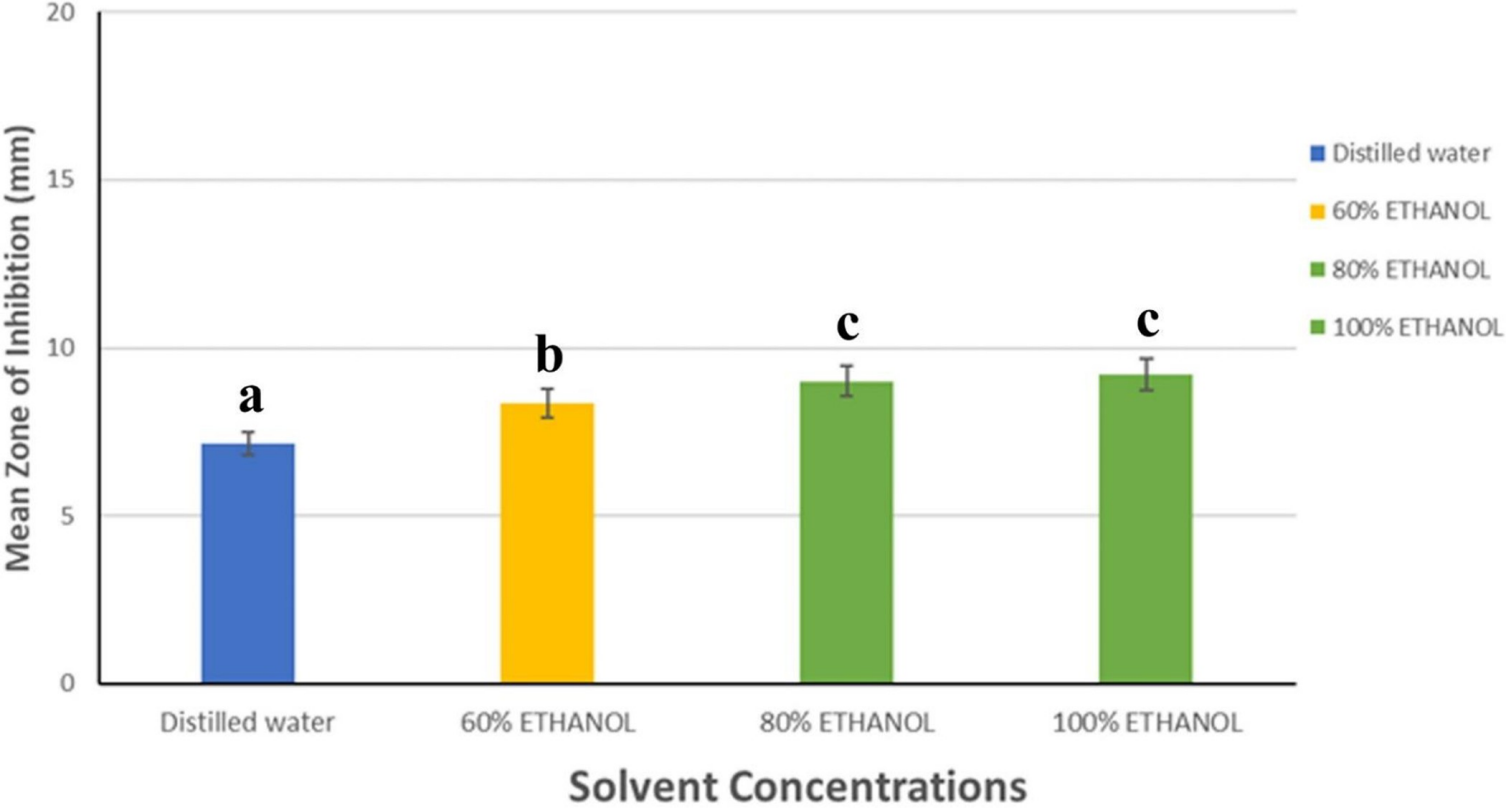
Tukey’s post hoc pairwise comparisons of mean zones of inhibition for different solvent concentrations and 95% confidence interval of tested groups.

The variables showed parametric distribution and thus one-way ANOVA was used to test the antibacterial effect across the plants’ extracts and solvent concentrations against the bacterial strains followed by Tukey’s post hoc for pairwise comparison between the tested groups (Figs 2–5). An ANOVA indicated the extract’s statistically significant antibacterial effect against the bacterial strains, P value < 0001. Tukey’s Post Hoc Pairwise Comparisons of the mean zone of inhibition across different plant extracts revealed that the root extract, with a mean zone of inhibition of 10.09 mm, is significantly more effective than all the other extracts (Fermented fruit, Dried fruit, Fresh fruit, and leaf) (Fig 3). The mean ZoI of the groups, Fermented Fruit extract, Dried Fruit extract, and Fermented Fruit juice (negative control) showed no significant difference but had significantly greater antibacterial activity than the fresh fruit extract, leaf extract, and fresh fruit juice (negative control). The antibiotic (Ciprofloxacin) used as a positive control, shows the highest mean zone of inhibition at 28.11 mm (Fig 3).

**Fig 6:**
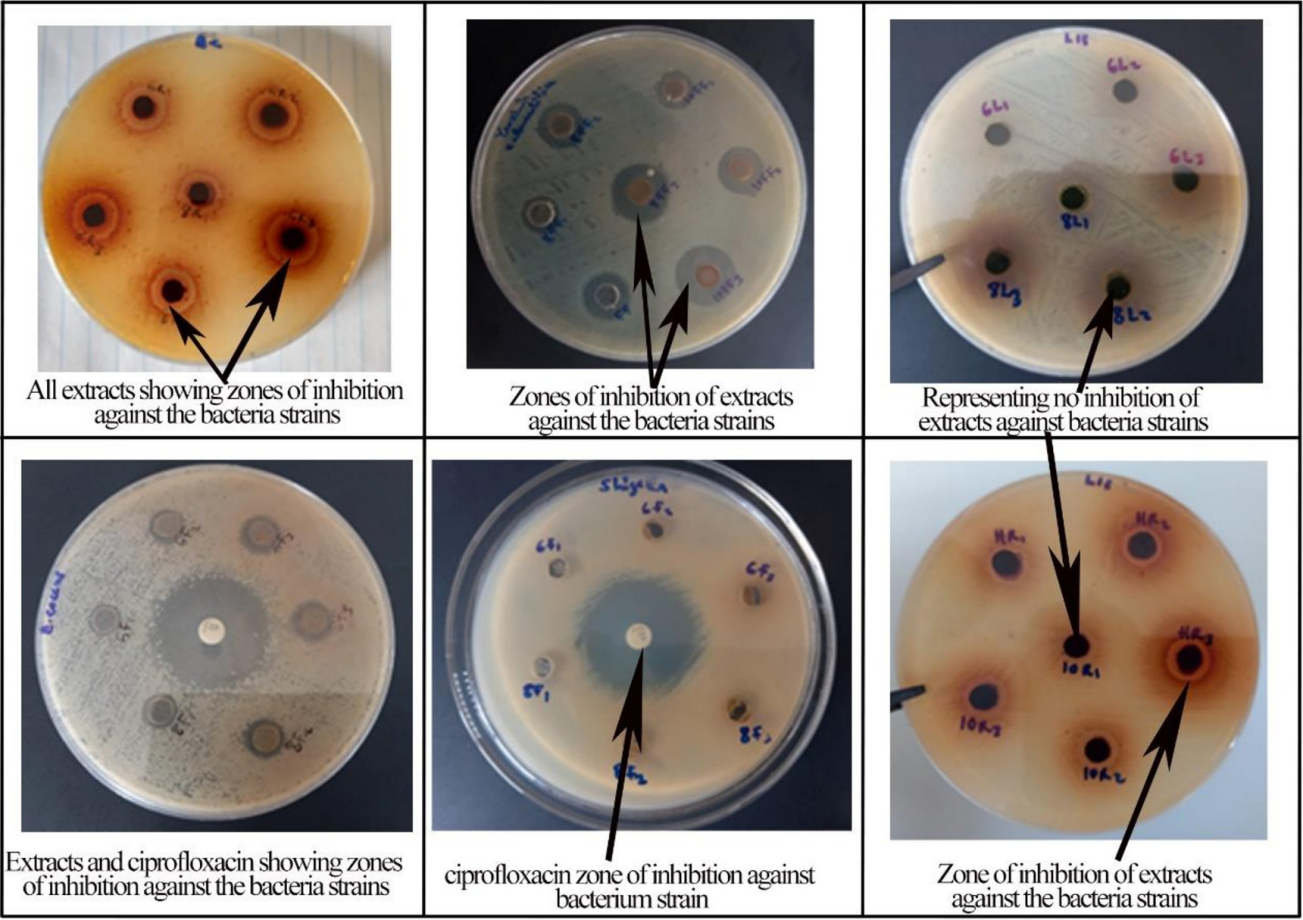
Zone inhibition of extracts and ciprofloxacin against some bacterial strains.

Again, Tukey’s post-hoc test was used to perform pairwise comparisons between the solvent concentrations (Figs 4 and 5). The results showed that the mean zone of inhibition for 100% ethanol was significantly higher than that of 60% ethanol (mean difference = 0.86819, p < 0.001) and dH_2_O (mean difference = 2.06720, p < 0.001). Similarly, 80% ethanol exhibited a significantly higher zone of inhibition compared to 60% ethanol (mean difference = 0.65851, p = 0.010) and dH_2_O (mean difference = 1.85753, p < 0.001). However, there was a statistically insignificant difference between the mean values of 100% ethanol and 80% ethanol (mean difference = 0.20968, p = 0.753) (Fig 5). Overall, the extract showed good inhibitory activity on almost all the microbes tested (Fig 6).

### 3.3 Activity index of plant extract

Table 2 compares the activity index of various plant (*M*. *citrifolia*) components to that of ciprofloxacin. In this current study, the activity index for all plant extracts in ethanol and dH_2_O was calculated and compared to that of the antibiotic (ciprofloxacin). Ethanol extracts had a higher activity index than dH_2_O and the root extract had the highest values recorded whereas the leaf had the lowest.

**Table 2:** Activity index of different plant extracts.

Organism	Fermented fruit (mm)		Dried fruit (mm)		Fresh Fruit (mm)		Root (mm)		Leaf (mm)	
	Ethanol	dH_2_O	Ethanol	dH_2_O	Ethanol	dH_2_O	Ethanol	dH_2_O	Ethanol	dH_2_O
*Y*. *enterocolitica*	0.36	0.3	0.36	0.26	0.31	0.19	0.26	0.19	0.19	0.2
*L*. *monocytogens*	0.47	0.28	0.45	0.35	0.36	0.28	0.5	0.41	0.25	0.25
*E*. *faecium*	0.36	0.27	0.43	0.36	0.38	0.29	0.48	0.36	0.23	0.23
*Campylobacter* spp.	0.31	0.25	0.29	0.22	0.34	0.26	0.62	0.39	0.22	0.22
*B*. *cereus*	0.33	0.31	0.43	0.34	0.36	0.26	0.46	0.4	0.30	0.23
*Shigella* spp.	0.39	0.32	0.30	0.23	0.23	0.23	0.23	0.23	0.23	0.23
*V*. *cholerae*	0.25	0.25	0.29	0.18	0.21	0.18	0.37	0.3	0.2	0.18
*Klebsiella* spp.	0.32	0.32	0.37	0.25	0.31	0.25	0.25	0.25	0.25	0.25
*E*. *coli*	-	-	0.31	0.23	0.24	0.23	0.31	0.23	0.23	0.23

(-) means no antibacterial activity of the extract was carried on against the bacteria.

## 4.0 Discussion

*Morinda citrifolia* has a long history as a medicinal plant and its traditional use in the treatment of several infectious ailments has grown tremendously in recent years. This has led to a parallel rise in studies on the biological activity and phytochemical components of the plant [23]. In the present study, the in vitro antibacterial activity of the five-plant extract (fermented fruit, dried fruit, fresh fruit, root, and leaf) of *M*. *citrifolia* using ethanol and dH_2_O as solvents on the eight (8) pathogenic bacteria indicates that the five-plant extracts were active not just on Gram-positive strains (*Listeria monocytogens*, *Bacillus cereus*, *Enterococcus faecium)* but as well on Gram-negative strains (*Klebsiella* spp., *Campylobacter* spp., *Vibrio cholerae*, *Yersinia enterocolitica*, *Shigella* spp., *E*. *coli* ATCC 25922). This observed difference is significant for each juice on the strains depending on the bacteria strain and the type of solvent extract (p < 0.001). The choice of these microorganisms for the experiments is associated with these bacteria being human pathogens commonly isolated in various hospitals in the Northern part of Ghana [24]. The findings from this study are in line with that of Sina et al [18] who demonstrated that *M*. *citrifolia* fruit extracts had an inhibition effect on the ten (10) pathogenic bacterial strains that were examined, five of which were Gram-positive bacteria (*Staphylococcus aureus*, *Staphylococcus epidermidis*, *Micrococcus luteus*, *Streptococcus oralis*, and *Enterococcus faecalis*) and five Gram-negative bacteria (*Pseudomonas aeruginosa*, *Proteus mirabilis*, *Proteus vulgaris*, *Escherichia coli*, *Escherichia coli*, and *Salmonella typhi*). Similarly, Srinivasahan & Durairaj [25] confirmed the antibacterial potential of *M*. *citrifolia* fruit extract against a range of bacterial strains (both Gram-positive and Gram-negative bacterial strains). The authors revealed that the presence of polyphenols, alkaloids, and glycosides are the secondary metabolites that exerted the inhibition force on the bacteria strains.

The differences in antibacterial efficacy between the various plant parts in the present study may be attributed to variations in their phytochemical composition. Ali et al [26] have discovered that diverse profiles of secondary metabolites, including iridoids, anthraquinones, and flavonoids, are present in the root, fruit, and leaf of *M*. *citrifolia*. These differences may be responsible for the plants’ varying antibacterial activity. In the present study, the mean ZoI for the plant extracts was found best in the root extract, followed by dried fruit, fermented fruit extract, and fresh fruit, and the least with the leaf extract for both ethanol and distilled water solvent extractions. It was observed that the root’s average inhibition diameter was either higher than or equal to all the other extracts and worked best in the 80% ethanol solvent against *Campylobacter* spp. (21.33 ± 1.80). More reports on the *M*. *citrifolia* fruit and leaf extracts have been reported to have more antibacterial activity with few on the root [27]. However, evidence from the present study revealed that the root extracts demonstrated significantly higher activity compared to the other plant parts of *M*. *citrifolia* (Fig 3). The higher efficacy of the root extracts may be attributed to the presence of higher amounts of flavonoids, terpenoids, alkaloids, and steroids, which are the secondary metabolites that exert the inhibition force on the bacteria strains [13,16]. Also, the findings agree with Soetan et al [28] who after employing methanolic extracts of leaf, root, and stem of *M*. *citrifolia*, demonstrated that the roots and leaves were the vegetal structures that supplied the extracts with a more potent antibacterial action against the bacterial strains. Their study further revealed that the roots’ sterols were the elements that had the greatest impact on the antibacterial action, and therefore it is believed there were a greater amount of sterols and other bioactive compounds in the root of the *M*. *citrifolia* cultivar used which then accounted for the mechanism of the action recorded. In addition, Lv and colleagues confirmed that *M*. *citrifolia* root contains strong phytochemical compounds as they isolated three new anthraquinones together with 15 known phytochemical compounds [29]. However, leaf extracts showed the lowest performance among the plant extracts which is against the same study of Soetan et al [28].

Also, for all the leaf solvent extracts, no inhibition was recorded for *Listeria monocytogens*, *Enterococcus faecium*, *Campylobacter*, *Shigella*, *Klebsiella*, and the control (*E*. *coli*). Nevertheless, report has it the antibacterial activity of *M*. *citrifolia* on different plant parts including the leaf exhibited inhibitory force against various pathogens including *Staphylococcus aureus*, *Bacillus subtilis*, *Escherichia coli*, *Pseudomonas aeruginosa*, *Salmonella* and *Shigella* because it contains a higher phenolic compound [19]. A study by Usha et al [30] revealed that *M*. *citrifolia* leaf extracts showed inhibitory activity against all the tested organism (*E*. *coli*, *Staphylococcus aureus*, *Candida albicans*, and *Aspergillus niger*) using solvents such as ethanol and water. Recently, Das and Aruna demonstrated an aqueous leaf extract of *M*. *citrifolia* having maximum antibacterial activity against *Escherichia coli* and *Pseudomonas aeruginosa* [31]. The lowest performance of the leaf extracts may be due to the drying and the extraction process which might have affected the potent phytoconstituents including the phenolic content of the leaf extracts [32].

More so, compared to fresh fruit extracts, the fermented fruit extract had inhibition against all the tested pathogens including *Shigella* and *Klebsiella* (Table 1). Additionally, a similar observation of significantly higher activity was found in the control comparisons for the fermented extracts. This suggests that fermentation increases the secondary metabolites such as polyphenols, alkaloids, and glycosides present in the fruit juice which are responsible for the antibacterial activity [25]. However, the observed differences between the fresh and fermented fruit extracts did not agree with the findings of Sina et al [18] who revealed that the inhibition diameter of fresh juice is either greater than or equal to that of fermented juice for each of the ten bacteria strains used. The observed differences may result from the fermentation processes which might increase the ethanol level of the fermented fruit juice and hence dissolve most of the secondary metabolites which accounts for the action recorded [33].

*Enterococcus faecium*, *Campylobacter* spp., and *Bacillus cereus* were found to be most sensitive to all plant extracts in the present study showing that *M*. *citrifolia* could be best used in the treatment of infectious diseases caused by such bacteria [18]. Conversely, *Shigella*, *Klebsiella* spp., and *E*. *coli* were less sensitive to the plant extracts in the present study and all bacteria strains showed susceptibility to the root extract except *Klebsiella* spp., and *Shigella* spp. which could not be inhibited by root, fresh fruit, and leaf extracts. The results were consistent with that of Sunder et al [27] who also reported that *Klebsiella* spp., *Salmonella* spp., and *Staphylococcus aureus* were not inhibited by all of the *M*. *citrifolia* (fruit, leaf, and seed) extracts. Also, Jayaraman et al [34] revealed that the ethyl acetate extract of *M*. *citrifolia* fruit was effective against most of the microorganisms except *Psedomonas aeruginosa* and *Klebsiella pneumoniae*. The reduced susceptibility of *Klebsiella* spp. and other Gram-negative bacteria may be due to resistant strains that have developed as *Klebsiella* spp. is recognized to be the primary cause of the majority of severe Gram-negative infections in healthcare environments [35]. Again, Gnimatin and his team revealed from clinical bacteriology diagnostics studies that *Klebsiella* spp. *Moraxella* spp. and *Escherichia* spp. were the top three multidrug-resistant bacteria causing infections in the Northern Region of Ghana necessitating the development of more potent medications to tackle these pathogens [24]. Nonetheless, the fact that this strain of *Klebsiella* spp. was inhibited by the Fermented fruit extracts suggests that *M*. *citrifolia* extracts may hold promising antibacterial properties, particularly against strains known for antibiotic resistance suggesting it as a potential alternative treatment option in the fight against multidrug-resistant bacteria.

The present study also investigated the influence of solvent polarity on the antibacterial activity of the *M*. *citrifolia* extracts. The choice of solvents (distilled water, 60%, 80%, and 100% ethanol) was based on their varying polarities, allowing for the extraction of a wide range of phytochemicals [36]. The solvents utilized affected the antibacterial activity of the various extracts. Selvam and colleagues reported on the antibacterial activity of the different extracts of *M*. *citrifolia* fruit powder using acetone, chloroform, methanol, and ethanol solvents where the researchers studied that, solvents extraction had inhibition against *Staphylococcus aureus*, *E*. *coli*, and *Proteus vulgaris* in nutrient agar medium when compared to standard levofloxacin [23]. In the present study, the outcomes obtained from the agar well diffusion assay showed that there was an increasing effect on microbial growth inhibition with increasing concentration of the ethanol extract. The highest result was obtained with 100% ethanol extract followed by 80% ethanol, 60% ethanol, and dH_2_O extracts. In a current study done in 2023, Thao et al. [37] also explored the antibacterial activity of *Morinda citrifolia* ethanol extracts and found that the inhibition zones increased with increasing ethanol concentrations and thus recorded higher values in higher concentrations as in this study. However, the lack of a significant difference in the zones of inhibition between 100% ethanol and 80% ethanol suggests that while 100% ethanol may not be significantly more effective than 80% ethanol, it is notably more potent than 60% ethanol and dH_2_O which showed significant differences. This indicates from the study that 80% ethanol could be a preferable choice for applications requiring antibacterial activity whereas concentrations below 80% may not be sufficient for optimal antibacterial action. This was in line with a recent study conducted by Soyer et al [38] who revealed that the usage of concentrations of 80% or 85% ethanol rather than 70% in the disinfection process in healthcare facilities will be more effective in the elimination of pathogens. The fact that ethanol extracts exhibit a greater level of antibacterial activity than those of distilled water suggests that the active ingredients responsible for the bactericidal action are more soluble in organic solvents (ethanol) [39]. The combined activity of numerous chemical components, rather than a single component, led to the observed efficiency of the plant extracts. The polarity of the molecules affects how easily the antibacterial compounds may be extracted. The polarity of the solvents influences the difference in antibacterial activity between the various solvent extracts. Due to the stronger polarity of ethanol than distilled water, ethanol tends to dissolve multiple compounds from the plant which is reflected in the frequent use of ethanol in the extraction of antimicrobial compounds [40,41]. The results were confirmed after calculating for activity index (Table 2) for both ethanol and distilled water against ciprofloxacin (CIP) where ethanol extracts had the highest values compared to those of distilled water.

The activity index was done to assess the relative effectiveness of the plant extracts against the different bacterial isolates to the widely used ciprofloxacin [20]. The results obtained demonstrated that the plant extracts were midway equal to the antibiotic inhibition activity (1 ≤ 1) as seen in Table 2. The highest values were recorded by the root extract against the bacteria strains which confirmed its effectiveness as compared to the other extracts in the present study. This observation is very relevant and future-looking because it may be possible to create therapeutic drugs from the active components that will be effective against multi-drug resistant organisms. The overall outcome of the antibacterial activity from various *M*. *citrifolia* plant parts showed that the best activity was found in root extract and was more sensitive in the 80% ethanol against the *Campylobacter* spp. which may be further developed for the extraction of antibacterial compounds from this plant component. The least activity was obtained in the leaf. Antibacterial compounds could be extracted from any component of the plant since they showed inhibition against almost all the bacteria strains. The considerable difference in activity between the plant extracts and CIP (Antibiotic) highlights the need for ongoing research to optimize the use of plant-based treatments in combating bacterial infections.

Even though we discovered an inhibitory impact against the pathogenic bacteria, the current study’s shortcoming was that the pathogens under investigation did not provide much information on strains of multidrug resistance that are widespread and also, lack of evaluation of the specific phytochemicals responsible for the inhibitory effects seen. Variability in extraction yields, which might result from variations in solvent type and concentration, is also one possible study constraint. Subsequently, future research ought to be conducted to validate and identify the secondary metabolites that demonstrated the antibacterial action and assess their efficacy against a more comprehensive panel of clinically relevant, drug-resistant bacterial isolates. The potent phytoconstituents that are present in the plant extract are believed to be responsible for the antibacterial activity that was seen. This could be a hint of a large potential for extracting purer compounds as well as better antibacterial compounds.

## 5.0 Conclusion

In this study, the root extract of *M*. *citrifolia* exhibited the most effective antibacterial activities against tested pathogens among the various plant parts examined, particularly in 80% ethanol extract. The type of plant part and the solvent used both had an impact on the extracts’ efficacy. The extracts with higher ethanol concentrations (100% and 80%) typically outperformed dH2O extracts. Despite the limitations, the findings suggest that *M*. *citrifolia* has significant potential as a source of antibacterial agents against both gram-positive and gram-negative bacteria strains. As multidrug-resistant bacteria continue to pose a significant public health challenge, the incorporation of plant-based therapeutics may offer efficient ways to supplement conventional antibiotic treatments. Future studies should focus on standardizing extraction methods, evaluating plant extracts against a wider range of clinically relevant drug-resistant bacterial isolates, isolation and characterization the active compounds responsible for the observed antibacterial effects, and in vivo studies investigations, or clinical trials of the plant extracts to assess the extracts’ potential for therapeutic use.

## Supporting information

S1 Table Antibacterial activity of different extracts of *M*. *citrifolia* against the pathogenic bacteria in different concentrations. (DOCX)
