# Supplementary material for: In vitro antibacterial activity of Morinda citrifolia extracts against eight pathogenic bacteria species
Source: PLoS One. 2024 Oct 30;19(10):e0313003. doi: 10.1371/journal.pone.0313003 (PMC11524514; doi:10.1371/journal.pone.0313003)
Supplement: S1 Table — (DOCX) [file pone.0313003.s001.docx]

| **PLANT EXTRACTS** | ***YERSINIA*** | ***LISTERIA*** | ***ENTERO***  ***COCCUS*** | ***CAMPY***  ***LOBACTER*** | ***B. CEREUS*** | ***SHIGELLA*** | ***V. CHOLERAE*** | ***KLEB***  ***SIELLA*** | ***E. COLI*** |
| --- | --- | --- | --- | --- | --- | --- | --- | --- | --- |
| **10FF1** | 10 | 6 | 8 | 7 | 6 | 8 | 7 | 7 | - |
| **10FF1** | 11 | 6 | 8 | 8 | 6 | 8 | 7 | 7 | - |
| **10FF1** | 10 | 6 | 8 | 7 | 6 | 8 | 7 | 7 | - |
| **10FF2** | 13 | 15 | 10 | 10 | 8 | 11 | 10 | 9 | - |
| **10FF2** | 14 | 15 | 10 | 10 | 8 | 12 | 10 | 9 | - |
| **10FF2** | 14 | 15 | 10 | 11 | 8 | 11 | 10 | 9 | - |
| **10FF3** | 15 | 20 | 13 | 9 | 11 | 14 | 11 | 10 | - |
| **10FF3** | 16 | 18 | 13 | 10 | 12 | 14 | 11 | 10 | - |
| **10FF3** | 15 | 20 | 13 | 9 | 11 | 14 | 11 | 10 | - |
| **8FF1** | 10 | 6 | 10 | 6 | 6 | 7 | 6 | 6 | - |
| **8FF1** | 9 | 6 | 8 | 6 | 6 | 7 | 6 | 6 | - |
| **8FF1** | 10 | 6 | 10 | 6 | 6 | 7 | 6 | 6 | - |
| **8FF2** | 12 | 6 | 9 | 10 | 12 | 10 | 9 | 7 | - |
| **8FF2** | 12 | 6 | 9 | 10 | 11 | 10 | 9 | 7 | - |
| **8FF2** | 12 | 6 | 9 | 10 | 11 | 10 | 10 | 7 | - |
| **8FF3** | 15 | 10 | 11 | 11 | 13 | 14 | 10 | 10 | - |
| **8FF3** | 14 | 10 | 10 | 12 | 11 | 14 | 10 | 10 | - |
| **8FF3** | 14 | 10 | 11 | 11 | 11 | 14 | 10 | 10 | - |
| **6FF1** | 7 | 6 | 6 | 6 | 6 | 7 | 6 | 6 | - |
| **6FF1** | 6 | 6 | 6 | 6 | 6 | 7 | 6 | 6 | - |
| **6FF1** | 7 | 6 | 6 | 6 | 6 | 7 | 6 | 6 | - |
| **6FF2** | 9 | 6 | 7 | 7 | 6 | 10 | 7 | 6 | - |
| **6FF2** | 9 | 6 | 7 | 7 | 6 | 10 | 7 | 6 | - |
| **6FF2** | 9 | 6 | 7 | 7 | 6 | 10 | 7 | 6 | - |
| **6FF3** | 11 | 27 | 10 | 9 | 10 | 11 | 9 | 7 | - |
| **6FF3** | 12 | 25 | 10 | 9 | 9 | 11 | 9 | 7 | - |
| **6FF3** | 11 | 27 | 10 | 9 | 9 | 11 | 9 | 8 | - |
| **HFF1** | 6 | 6 | 6 | 6 | 6 | 6 | 6 | 6 | - |
| **HFF1** | 6 | 6 | 6 | 6 | 6 | 6 | 6 | 6 | - |
| **HFF1** | 6 | 6 | 6 | 6 | 6 | 6 | 6 | 6 | - |
| **HFF2** | 10 | 8 | 6 | 6 | 8 | 9 | 6 | 6 | - |
| **HFF2** | 10 | 8 | 6 | 6 | 7 | 9 | 6 | 6 | - |
| **HFF2** | 10 | 8 | 6 | 6 | 8 | 9 | 6 | 6 | - |
| **HFF3** | 13 | 6 | 9 | 9 | 10 | 10 | 8 | 9 | - |
| **HFF3** | 14 | 6 | 9 | 9 | 10 | 11 | 8 | 8 | - |
| **HFF3** | 13 | 6 | 9 | 9 | 10 | 10 | 8 | 9 | - |
| **10DF1** | 10 | 8 | 9 | 9 | 9 | 8 | 9 | 9 | - |
| **10DF1** | 9 | 8 | 9 | 9 | 9 | 8 | 9 | 9 | - |
| **10DF1** | 10 | 8 | 9 | 9 | 9 | 8 | 9 | 9 | - |
| **10DF2** | 12 | 11 | 11 | 11 | 11 | 8 | 12 | 9 | - |
| **10DF2** | 12 | 11 | 11 | 11 | 11 | 8 | 12 | 9 | - |
| **10DF2** | 12 | 11 | 11 | 11 | 11 | 8 | 12 | 9 | - |
| **10DF3** | 15 | 15 | 13 | 12 | 12 | 11 | 13 | 11 | 10 |
| **10DF3** | 15 | 15 | 13 | 12 | 12 | 11 | 13 | 11 | 10 |
| **10DF3** | 15 | 15 | 13 | 12 | 12 | 11 | 13 | 11 | 10 |
| **8DF1** | 10 | 8 | 10 | 6 | 7 | 7 | 8 | 9 | - |
| **8DF1** | 10 | 8 | 10 | 6 | 7 | 7 | 8 | 9 | - |
| **8DF1** | 10 | 8 | 10 | 6 | 7 | 7 | 8 | 9 | - |
| **8DF2** | 12 | 10 | 11 | 6 | 13 | 7 | 12 | 9 | - |
| **8DF2** | 12 | 10 | 11 | 6 | 13 | 7 | 12 | 9 | - |
| **8DF2** | 12 | 10 | 11 | 6 | 13 | 7 | 12 | 9 | - |
| **8DF3** | 13 | 14 | 12 | 9 | 15 | 8 | 14 | 9 | 8 |
| **8DF3** | 14 | 14 | 12 | 9 | 15 | 8 | 14 | 9 | 8 |
| **8DF3** | 14 | 14 | 12 | 9 | 15 | 8 | 14 | 9 | 8 |
| **6DF1** | 9 | 8 | 9 | 6 | 9 | 7 | 6 | 8 | - |
| **6DF1** | 9 | 8 | 9 | 6 | 9 | 7 | 6 | 8 | - |
| **6DF1** | 9 | 8 | 9 | 6 | 9 | 7 | 6 | 8 | - |
| **6DF2** | 10 | 9 | 10 | 6 | 11 | 7 | 6 | 8 | - |
| **6DF2** | 10 | 9 | 10 | 6 | 11 | 7 | 6 | 8 | - |
| **6DF2** | 10 | 9 | 10 | 6 | 12 | 7 | 6 | 8 | - |
| **6DF3** | 12 | 12 | 12 | 6 | 12 | 7 | 8 | 8 | 6 |
| **6DF3** | 12 | 12 | 12 | 6 | 12 | 7 | 8 | 8 | 6 |
| **6DF3** | 12 | 12 | 12 | 6 | 12 | 7 | 8 | 8 | 6 |
| **HDF1** | 7 | 8 | 8 | 6 | 7 | 6 | 6 | 6 | - |
| **HDF1** | 7 | 8 | 8 | 6 | 7 | 6 | 6 | 6 | - |
| **HDF1** | 7 | 8 | 8 | 6 | 7 | 6 | 6 | 6 | - |
| **HDF2** | 9 | 8 | 9 | 6 | 9 | 6 | 6 | 6 | - |
| **HDF2** | 8 | 8 | 9 | 6 | 9 | 6 | 6 | 6 | - |
| **HDF2** | 9 | 8 | 9 | 6 | 9 | 6 | 6 | 6 | - |
| **HDF3** | 10 | 9 | 10 | 6 | 10 | 6 | 6 | 6 | 6 |
| **HDF3** | 9 | 9 | 10 | 6 | 10 | 6 | 6 | 6 | 6 |
| **HDF3** | 9 | 9 | 10 | 6 | 10 | 6 | 6 | 6 | 6 |
| **10F1** | 8 | 8 | 8 | 9 | 7 | 6 | 8 | 6 | - |
| **10F1** | 8 | 8 | 8 | 9 | 7 | 6 | 8 | 6 | - |
| **10F1** | 8 | 8 | 8 | 9 | 7 | 6 | 8 | 6 | - |
| **10F2** | 11 | 8 | 10 | 9 | 9 | 6 | 8 | 8 | - |
| **10F2** | 11 | 8 | 10 | 9 | 9 | 6 | 8 | 8 | - |
| **10F2** | 11 | 8 | 10 | 9 | 9 | 6 | 8 | 8 | - |
| **10F3** | 14 | 12 | 13 | 11 | 13 | 6 | 12 | 10 | 7 |
| **10F3** | 13 | 12 | 13 | 11 | 13 | 6 | 12 | 10 | 7 |
| **10F3** | 13 | 12 | 13 | 11 | 13 | 6 | 12 | 10 | 7 |
| **8F1** | 7 | 6 | 8 | 9 | 8 | 6 | 6 | 6 | - |
| **8F1** | 7 | 6 | 8 | 9 | 8 | 6 | 6 | 6 | - |
| **8F1** | 7 | 6 | 8 | 9 | 8 | 6 | 6 | 6 | - |
| **8F2** | 9 | 8 | 9 | 9 | 9 | 6 | 6 | 8 | - |
| **8F2** | 9 | 8 | 9 | 9 | 9 | 6 | 6 | 8 | - |
| **8F2** | 9 | 8 | 9 | 9 | 9 | 6 | 6 | 8 | - |
| **8F3** | 11 | 10 | 11 | 13 | 11 | 6 | 6 | 9 | 6 |
| **8F3** | 11 | 10 | 11 | 13 | 11 | 6 | 6 | 9 | 6 |
| **8F3** | 11 | 10 | 11 | 13 | 11 | 6 | 6 | 9 | 6 |
| **6F1** | 6 | 7 | 8 | 7 | 7 | 6 | 6 | 6 | - |
| **6F1** | 6 | 7 | 8 | 7 | 7 | 6 | 6 | 6 | - |
| **6F1** | 6 | 7 | 8 | 7 | 7 | 6 | 6 | 6 | - |
| **6F2** | 10 | 8 | 9 | 7 | 9 | 6 | 6 | 7 | - |
| **6F2** | 10 | 8 | 9 | 7 | 9 | 6 | 6 | 7 | - |
| **6F2** | 10 | 8 | 9 | 7 | 9 | 6 | 6 | 7 | - |
| **6F3** | 14 | 9 | 10 | 8 | 11 | 6 | 6 | 7 | 6 |
| **6F3** | 14 | 9 | 10 | 8 | 11 | 6 | 6 | 7 | 6 |
| **6F3** | 14 | 9 | 10 | 8 | 11 | 6 | 6 | 7 | 6 |
| **HF1** | 6 | 6 | 6 | 6 | 6 | 6 | 6 | 6 | - |
| **HF1** | 6 | 6 | 6 | 6 | 6 | 6 | 6 | 6 | - |
| **HF1** | 6 | 6 | 6 | 6 | 6 | 6 | 6 | 6 | - |
| **HF2** | 6 | 7 | 7 | 7 | 7 | 6 | 6 | 6 | - |
| **HF2** | 6 | 7 | 7 | 7 | 7 | 6 | 6 | 6 | - |
| **HF2** | 6 | 7 | 7 | 7 | 7 | 6 | 6 | 6 | - |
| **HF3** | 6 | 7 | 9 | 9 | 7 | 6 | 6 | 6 | 6 |
| **HF3** | 6 | 7 | 9 | 9 | 7 | 6 | 6 | 6 | 6 |
| **HF3** | 6 | 7 | 9 | 9 | 7 | 6 | 6 | 6 | 6 |
| **10R1** | 8 | 10 | 11 | 11 | 10 | 6 | 11 | 6 | - |
| **10R1** | 8 | 10 | 11 | 11 | 10 | 6 | 11 | 6 | - |
| **10R1** | 8 | 10 | 11 | 11 | 10 | 6 | 11 | 6 | - |
| **10R2** | 10 | 11 | 11 | 12 | 10 | 6 | 10 | 6 | - |
| **10R2** | 10 | 11 | 11 | 12 | 10 | 6 | 10 | 6 | - |
| **10R2** | 10 | 11 | 11 | 12 | 10 | 6 | 10 | 6 | - |
| **10R3** | 11 | 11 | 11 | 12 | 10 | 6 | 11 | 6 | 6 |
| **10R3** | 11 | 11 | 11 | 12 | 10 | 6 | 11 | 6 | 6 |
| **10R3** | 11 | 11 | 11 | 12 | 10 | 6 | 11 | 6 | 6 |
| **8R1** | 8 | 12 | 12 | 19 | 12 | 6 | 11 | 6 | - |
| **8R1** | 8 | 12 | 12 | 19 | 12 | 6 | 11 | 6 | - |
| **8R1** | 8 | 12 | 12 | 19 | 12 | 6 | 11 | 6 | - |
| **8R2** | 8 | 13 | 13 | 22 | 12 | 6 | 14 | 6 | - |
| **8R2** | 8 | 13 | 13 | 22 | 12 | 6 | 14 | 6 | - |
| **8R2** | 8 | 13 | 13 | 22 | 12 | 6 | 14 | 6 | - |
| **8R3** | 8 | 13 | 14 | 23 | 13 | 6 | 15 | 6 | 12 |
| **8R3** | 8 | 13 | 14 | 23 | 13 | 6 | 15 | 6 | 12 |
| **8R3** | 8 | 13 | 14 | 23 | 13 | 6 | 15 | 6 | 12 |
| **6R1** | 7 | 11 | 12 | 14 | 12 | 6 | 13 | 6 | - |
| **6R1** | 7 | 11 | 12 | 14 | 12 | 6 | 13 | 6 | - |
| **6R1** | 7 | 11 | 12 | 14 | 12 | 6 | 13 | 6 | - |
| **6R2** | 7 | 12 | 13 | 20 | 14 | 6 | 14 | 6 | - |
| **6R2** | 7 | 12 | 13 | 20 | 14 | 6 | 14 | 6 | - |
| **6R2** | 7 | 12 | 13 | 20 | 14 | 6 | 14 | 6 | - |
| **6R3** | 7 | 13 | 13 | 22 | 14 | 6 | 15 | 6 | 6 |
| **6R3** | 7 | 13 | 13 | 22 | 14 | 6 | 15 | 6 | 6 |
| **6R3** | 7 | 13 | 13 | 22 | 14 | 6 | 15 | 6 | 6 |
| **HR1** | 6 | 8 | 9 | 10 | 10 | 6 | 10 | 6 | - |
| **HR1** | 6 | 8 | 9 | 10 | 10 | 6 | 10 | 6 | - |
| **HR1** | 6 | 8 | 9 | 10 | 10 | 6 | 10 | 6 | - |
| **HR2** | 6 | 10 | 9 | 10 | 10 | 6 | 10 | 6 | - |
| **HR2** | 6 | 10 | 9 | 10 | 10 | 6 | 10 | 6 | - |
| **HR2** | 6 | 10 | 9 | 10 | 10 | 6 | 10 | 6 | - |
| **HR3** | 6 | 11 | 10 | 12 | 11 | 6 | 11 | 6 | 6 |
| **HR3** | 6 | 11 | 10 | 12 | 11 | 6 | 11 | 6 | 6 |
| **HR3** | 6 | 11 | 10 | 12 | 11 | 6 | 11 | 6 | 6 |
| **10L1** | 6 | 6 | 6 | 6 | 8 | 6 | 7 | 6 | - |
| **10L1** | 6 | 6 | 6 | 6 | 8 | 6 | 7 | 6 | - |
| **10L1** | 6 | 6 | 6 | 6 | 8 | 6 | 7 | 6 | - |
| **10L2** | 6 | 6 | 6 | 6 | 9 | 6 | 7 | 6 | - |
| **10L2** | 6 | 6 | 6 | 6 | 9 | 6 | 7 | 6 | - |
| **10L2** | 6 | 6 | 6 | 6 | 9 | 6 | 7 | 6 | - |
| **10L3** | 6 | 6 | 6 | 6 | 11 | 6 | 9 | 6 | 6 |
| **10L3** | 6 | 6 | 6 | 6 | 11 | 6 | 9 | 6 | 6 |
| **10L3** | 6 | 6 | 6 | 6 | 11 | 6 | 9 | 6 | 6 |
| **8L1** | 6 | 6 | 6 | 6 | 7 | 6 | 6 | 6 | - |
| **8L1** | 6 | 6 | 6 | 6 | 7 | 6 | 6 | 6 | - |
| **8L1** | 6 | 6 | 6 | 6 | 7 | 6 | 6 | 6 | - |
| **8L2** | 6 | 6 | 6 | 6 | 7 | 6 | 6 | 6 | - |
| **8L2** | 6 | 6 | 6 | 6 | 7 | 6 | 6 | 6 | - |
| **8L2** | 6 | 6 | 6 | 6 | 7 | 6 | 6 | 6 | - |
| **8L3** | 6 | 6 | 6 | 6 | 7 | 6 | 6 | 6 | 6 |
| **8L3** | 6 | 6 | 6 | 6 | 7 | 6 | 6 | 6 | 6 |
| **8L3** | 6 | 6 | 6 | 6 | 7 | 6 | 6 | 6 | 6 |
| **6L1** | 6 | 6 | 6 | 6 | 7 | 6 | 6 | 6 | - |
| **6L1** | 6 | 6 | 6 | 6 | 7 | 6 | 6 | 6 | - |
| **6L1** | 6 | 6 | 6 | 6 | 7 | 6 | 6 | 6 | - |
| **6L2** | 6 | 6 | 6 | 6 | 7 | 6 | 6 | 6 | - |
| **6L2** | 6 | 6 | 6 | 6 | 7 | 6 | 6 | 6 | - |
| **6L2** | 6 | 6 | 6 | 6 | 7 | 6 | 6 | 6 | - |
| **6L3** | 6 | 6 | 6 | 6 | 6 | 6 | 6 | 6 | - |
| **6L3** | 6 | 6 | 6 | 6 | 6 | 6 | 6 | 6 | - |
| **6L3** | 6 | 6 | 6 | 6 | 6 | 6 | 6 | 6 | - |
| **HL1** | 6 | 6 | 6 | 6 | 6 | 6 | 6 | 6 | - |
| **HL1** | 6 | 6 | 6 | 6 | 6 | 6 | 6 | 6 | - |
| **HL1** | 6 | 6 | 6 | 6 | 6 | 6 | 6 | 6 | - |
| **HL2** | 6 | 6 | 6 | 6 | 6 | 6 | 6 | 6 | - |
| **HL2** | 6 | 6 | 6 | 6 | 6 | 6 | 6 | 6 | - |
| **HL2** | 6 | 6 | 6 | 6 | 6 | 6 | 6 | 6 | - |
| **HL3** | 7 | 6 | 6 | 6 | 6 | 6 | 6 | 6 | 6 |
| **HL3** | 7 | 6 | 6 | 6 | 6 | 6 | 6 | 6 | 6 |
| **HL3** | 7 | 6 | 6 | 6 | 6 | 6 | 6 | 6 | 6 |
| **CIP 1** | 33 | 25 | 26 | 29 | 26 | 27 | 35 | 25 | 27 |
| **CIP 2** | 33 | 25 | 26 | 29 | 26 | 27 | 35 | 25 | 27 |
| **CIP 3** | 33 | 25 | 26 | 29 | 26 | 27 | 35 | 25 | 27 |
| **FRESH FRUIT 1** | 6 | 6 | 6 | 6 | 6 | 6 | 6 | 6 | - |
| **FRESH FRUIT 2** | 6 | 6 | 6 | 6 | 6 | 6 | 6 | 6 | - |
| **FRESH FRUIT 3** | 6 | 6 | 6 | 6 | 6 | 6 | 6 | 6 | - |
| **FERMENTED FRUIT 1** | 10 | 6 | 16 | 9 | 6 | 10 | 9 | 6 | - |
| **FERMENTED FRUIT 2** | 11 | 6 | 17 | 9 | 6 | 10 | 9 | 6 | - |
| **FERMENTED FRUIT 3** | 10 | 6 | 17 | 9 | 6 | 10 | 9 | 6 | - |

**KEY**

**10 = 100% ETHANOL EXTRACT**

**8 = 80% ETHANOL EXTRACT**

**6 = 60% ETHANOL EXTRACT**

**H = WATER EXTRACT**

**FF = FERMENTED FRUIT**

**DF = DRIED FRUIT EXTRACT**

**F = FRUIT JUICE EXTRACT**

**R = ROOT EXTRACT**

**L = LEAF EXTRACT**

**1 – FIRST CONCENTRATION**

**2 – SECOND CONCENTRATION**

**3 – THIRD CONCENTRATION**

**(-) = No antibacterial activity was carried out**
